# Factors Influencing Compliance and Health Seeking Behaviour for Hypertension in Mukono and Buikwe in Uganda: A Qualitative Study

**DOI:** 10.1155/2018/8307591

**Published:** 2018-04-26

**Authors:** Geofrey Musinguzi, Sibyl Anthierens, Fred Nuwaha, Jean-Pierre Van Geertruyden, Rhoda K. Wanyenze, Hilde Bastiaens

**Affiliations:** ^1^Department of Disease Control and Environmental Health, School of Public Health, Makerere University, Kampala, Uganda; ^2^Primary and Interdisciplinary Care, University of Antwerp, Antwerp, Belgium; ^3^International Health, University of Antwerp, Antwerp, Belgium

## Abstract

**Background and Methods:**

Hypertension is a global public health challenge and a leading risk factor for cardiovascular morbidity and mortality. Hypertension control rates are low worldwide, and delay in seeking care is associated with increased mortality.

**Methods:**

In a qualitative study, known hypertensive patients were interviewed to explore factors influencing compliance and health seeking behaviour (HSB). Data was analyzed following a semantic thematic analysis approach.

**Results:**

Patients sought various channels of care for their hypertension. Self-medication and access to antihypertensive drugs with or without prescription were common as well as use of herbal remedies. Regular monitoring of blood pressure was not a common practice. Factors influencing HSB were related to health systems and the patient socioeconomic and structural environment. The main system issues were related to availability and attitudes of staff and shortage of supplies and medicines. The patient factors were related to awareness, perceived severity, perceived effectiveness of therapy, adverse effects, and perceived fears of lifelong dependence on medicines. The patient socioeconomic status played a role as did the marketing of traditional medicine.

**Conclusion:**

Patients seek varied channels of care for their hypertension. Strategies to address the multifactorial dimensions that affect HSB are needed to improve hypertension control in this population.

## 1. Introduction

Hypertension is a leading risk factor for cardiovascular morbidity and mortality worldwide [1]. A comparative risk analysis of the burden of disease and injury associated with 67 risk factors revealed that hypertension (also known as high blood pressure) is the leading risk factor for mortality with 7% of deaths attributable to it [1]. It is predicted that, by 2025, the number of adults with hypertension will increase by about 60% to a total of 1.56 billion and most of the cases will occur in low and middle income countries (LMIC) [2]. Urbanization and the epidemiological transition characterised by an aging population, physical inactivity, obesity, increasing alcohol consumption, and high salt intake are contributing to the increasing rates of hypertension in LMIC [3]. Hypertension is a “silent killer” [4] and data suggests that hypertensive heart disease could be the most common form of cardiovascular disease (CVD) in Africa [5]. Unfortunately, most cases of hypertension are asymptomatic and as a result, hypertensive patients often seek medical attention late or when they have developed complications such as strokes, heart attacks, heart failure, and kidney failure [3]. In their publication, Cruickshank et al. on the rule of halves reported that 50% of the hypertensives are known, of the known hypertensives, half are on treatment and of those on treatment, half are controlled [5]. The World Health Organization (WHO) projected that, over the next ten years, Africa will experience the largest increase in death rates from CVDs. Consequently, the negative economic impact of CVDs will be felt more on the African continent [6] and the cost of handling chronic illness will lead many households to poverty [7].

Health seeking behaviour (HSB) has been defined as any activity undertaken by individuals who perceive themselves to have a health problem or to be ill for the purpose of finding an appropriate remedy. The desired HSB is responding to an illness by seeking help from a trained allopathic doctor in a recognized health care center [8]. It is well established that HSB is influenced by manifestation of symptoms [9, 10]. However, many hypertensive individuals may not be aware and may not have any symptoms to compel them to seek care. Even among those presenting with symptoms and those who are aware hypertensive, their HSB is suboptimal [11, 12]. Hypertension control remains very low [13] and attributable reasons revolve around noncompliance due to side effects of the medication, lack of information and support, difficulty obtaining the medication, poverty, low education, and poor access to health care [14–16]. In addition, health system deficiencies such as lack of antihypertensive medication, the physicians' inertia in treating hypertension, and long distance to the health facilities have been implicated in affecting HSBs and control of hypertension [13, 17, 18]. Delays in seeking care for hypertension are associated with increased mortality [19] and the benefits of early treatment and control are also well established [20–22].

Like most of Sub-Saharan Africa, Uganda's health care system was established with a greater orientation towards communicable diseases. It is only in the past 15 years that the NCD agenda started featuring in the national health strategic plan of the Ministry of Health [23]. The current health service delivery system has a number of gaps ranging from lack of preventive services for NCDs to lack of essential supplies for managing NCDs [24].

Over the last 20 years, Uganda has witnessed an increasingly aging population which has ushered in increasing rates of lifestyle-related chronic noncommunicable diseases [25]. Notably, increasing cases of obesity, diabetes mellitus, and hypertension, along with such complications as stroke, and heart diseases are reported [25]. Data from the Health management information system (HMIS) suggests that hypertension is the most reported NCD and community surveys also suggest that one in five of every adult ≥ 18 years have uncontrolled hypertension [26]. The detailed reasons for low levels of hypertension control and inadequate HSB in this setting are not well understood.

We explored compliance and HSBs for hypertension using qualitative approach in order to understand reasons for low levels of hypertension control. Understanding the HSB for hypertension is vitally important in designing programs for hypertension control and enhancing quality standards in healthcare delivery [8]. 

## 2. Methods

### 2.1. Setting and Design

From August to October 2014, we conducted a community descriptive qualitative study and performed semistructured individual interviews with 48 patients with known hypertension in Mukono and Buikwe districts in Uganda. According to the Uganda population and housing census area specific profiles, 78% and 72% of the population in Buikwe and Mukono, respectively, live in rural areas [27]. The Uganda National Health System is made up of the Public and the Private sectors. Uganda implements a level based healthcare delivery system with a referral framework from a lower facility (Health Center (HC) II, III, IV) through hospitals at the district, regional, or national level. Mukono and Buikwe districts combined have six (6) hospitals, 4 HCIV, 27 HCIIIs, and 57 HCIIs. Moreover, most of these facilities are ill-equipped to provide services for hypertension. For example, essential ingredients including diagnostic equipment, antihypertensive medicines, and personnel are scarce [28]. Secondly, conducting this study in this setting would provide incremental evidence to our previously conducted WHO Stepwise Approach to Chronic Disease Risk Factor Surveillance (STEPS) study [29]. In the STEPS study, we observed an unacceptably high prevalence rate of hypertension and suboptimal treatment and control [29].

### 2.2. Sampling

A purposive sample of patients with known hypertension was identified from the database gathered two years before [29]. The STEPS, conducted in 2012, enrolled 4653 study participants of whom 258 were hypertensive patients and aware of their hypertension. The data set for the 258 patients was organised by gender, residential status, and district to select patients for enrolment into the current study. To generate a wide range of experiences, a fairly big sample size (48) comprising of a varied group of patients (males' and females, urban and rural, Buikwe and Mukono districts) were interviewed. The final sample is comprised of equal number of patients drawn from both districts of Mukono and Buikwe, 17 males, and 24 rural residents. All participants consented except two males.

### 2.3. Data Collection

Data were collected by one of the lead investigators (GM) and three graduate research assistants who underwent a rigorous and extensive 5-day training to standardise data collection procedures. The training entailed a review of the study objectives, conduct of qualitative inquiries, blood pressure measurement using a validated automated digital sphygmomanometer (Omron model M3W) with an appropriate cuff size, interpersonal communication skills, ethics, confidentiality, and informed consent, seminars on one-on-one interviewing techniques, and detailed instructions on using the semistructured interview protocol. The use of semistructured interview guides (Appendix) enabled data gathering on background characteristics of the study patients, compliance, and HSBs for hypertension. Interviews were conducted at the participants' homes. Each interview lasted one hour on average after which the patients' blood pressure was measured thrice one minute apart. Measuring blood pressure after the interview allowed the patient to relax and also prevented any biased responses that would arise if measurements were done before the interviews. All interviews were audio-recorded with consent from each patient and the average of the three readings was used to estimate the patient blood pressure.

### 2.4. Ethics Statement

Ethical approval was granted by Makerere University School of Public Health Higher Degrees-Research and Ethics Committee and the Uganda National Council of Science and technology. Each participant provided written informed consent.

### 2.5. Data Management

All qualitative data were transcribed verbatim and then translated into English from the local language. Each transcript was reviewed by at least two research assistants and one of the investigators (GM) for content and completeness. Final transcripts were stored securely on pass-worded external drives and later exported to ATLAS.ti version 7, qualitative data management software for further management.

### 2.6. Data Analysis

To identify the factors influencing compliance and health seeking behaviours for hypertension, we analysed the data following a semantic approach in thematic analysis [30]. Using free coding options in ATLAS.ti, GM read each transcript and extracted relevant data to generate codes related to compliance and health seeking behaviours. The codes were reviewed to identify patterns within the data [31] and iteratively compared with the original transcripts. Following the review and with use of code families' options in ATLAS.ti, the codes were sorted into groups (to generate subthemes) and where appropriate were split or dropped or renamed if better subthemes were obtained. The sub-themes were further reanalysed to generate themes and overarching themes. For quality control, and to ensure that the interpretation was close to the content and to support reflexivity, the process of analysis and output at every stage of the analysis was regularly reviewed and discussed with SA and HB.

## 3. Results

### 3.1. Participants and Context

Table 1 shows study participants' characteristics and their current blood pressure profiles as measured at the time of the interviews.

As shown in Table 1, 48 patients were interviewed between August and October 2014. Of these, 17 were males, 24 were rural residents, 35 were married, and 23 had attained post-primary education. The median age was 54 (males 54, females 51.5). Very few patients, 8, had their hypertension under control (SBP < 140 mmHg and DBP < 90 mmHg). Majority (*n* = 20) were stage one hypertensive and sizable proportions were stage 2 (*n* = 11) and stage 3 (*n* = 9) hypertensive. Self-medication and access to antihypertensive drugs with or without prescription from drug outlets were common as well as use of herbal remedies. Some patients also reported engaging in life style interventions such as cessation of alcohol consumption, increased physical activity, and dietary modifications. Regular monitoring of blood pressure was not a common practice. Modern healthcare for hypertension was sought widely from all levels of health facilities including drug shops, pharmacies, private health clinics, health centers (II, III, and IV) and hospitals. Herbal remedies in use included garlic, ginger, pumpkin leaves* (essunsa)*, herbal mixtures (names unknown), African eggplant* (Katunkuma)*, Moringa,* Mumbwa*, spider plant* (ejjobyo)*, aloe vera* (kigaji)*, dried banana leaves, neem tree, chlorophyll extracts, human urine, Tianshi (Chinese herbal), and African night shade (Nakati).

### 3.2. Factors Influencing Compliance and Health Seeking Behaviour for Hypertension

**Figure 1:**
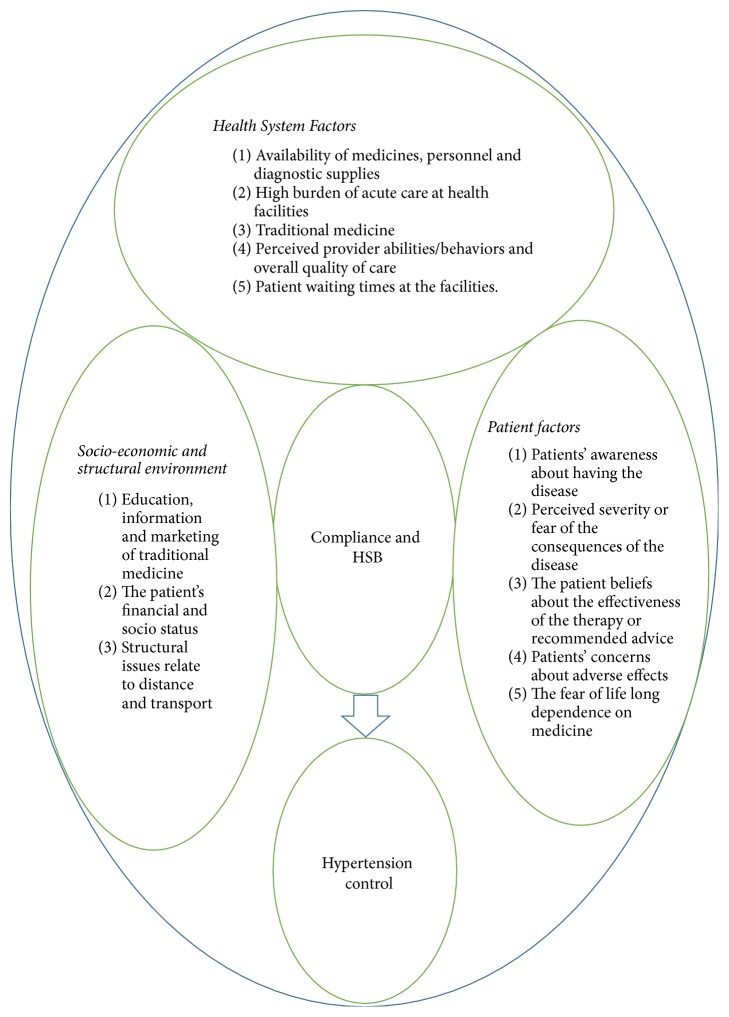
Factors influencing compliance and health seeking behaviours for hypertension among patients in Mukono and Buikwe Districts in Uganda.

The 3 thematic key issues influencing HSB for hypertension were related to the health system, the patient, and the broader socioeconomic and structural environment. Consequently, we organise the key findings (themes and subthemes) under each of these three overarching thematic areas.  Figure 1 illustrates the schema of the identified themes and subthemes.

#### 3.2.1. Health System Factors

Under the overarching theme of health system factors, five themes influencing compliance and HSBs were identified: (1) availability of medicines, personnel, and diagnostic supplies; (2) high burden of acute care at health facilities; (3) traditional medicines; (4) perceived provider abilities/behaviours and overall quality of care, and (5) patient waiting times at the facilities.

*(1) Availability of Medicine, Personnel, and Diagnostic Supplies*

*Medicines.* Patients noted that availability of or the assumption of [modern] medicines was readily available at health facilities and related costs played a key role in their seeking behaviours for hypertension. In private health facilities and private pharmacies, patients reported that antihypertensive drugs were readily available. However, high costs of these medicines were a deterrent to access. As such, they echoed that only those who had the money accessed the medicines as illustrated by the following patient in a local proverb.

*When you are a poor man, you don't prick a Swahili ban [local bread] or else you get beaten if you prick and don't buy. *(8 Bulamba-Bugungu_Male 1-*Buikwe, Rural*)

As a caveat to turn away from high costs, self-medication was commonly reported from drug outlets, which dispensed medicines with or without prescription and in any quantities depending on the patients' affordability. But even with this option, cost still deterred patients from access as illuminated by the following patient.

*Apart from the medicine he gave me. I failed to buy the medicine he had prescribed; even the last time I went to him; I failed to buy the medicine because it's expensive. One tablet costs 2,000 shs; and yet I had to swallow one per day for one month. I calculated and it was 60,000 shs; so I only swallowed the medicine he gave me; and then stopped. *(19 Kanyogoga_Male 2-*Buikwe, Urban*)

The costs of antihypertensive medicine were offset when the health facilities provided the medicine free of charge to patients. Indeed, patients attested that finding medicines at the health facilities was a motivation for their health seeking behaviours. However, lack of medicines had a negative implication on their compliance and health seeking behaviours. Patients expressed a sense of disappointment when they visited a health facility in anticipation of getting free medicines only to be given prescriptions to go and buy medicine elsewhere.

*I went there four times; the first two times I got the medicine; while the other two times I was told that there was no medicine. So I realized that I was wasting time and stopped going there. *(20 Kateete_Female1)

Another also echoed, “they gave me that medicine that I have told you [valium] and they told me to buy the other drugs since it was out of stock in the hospital. I didn't buy it; the truth is that the medicine was expensive” (12 Geregere Majjani_Female 1-*Buikwe, Urban*).

*Personnel.* Finding a service provider at a health facility was also critical in influencing health seeking behaviour of hypertensive patients. Patients were motivated to go to a health facility where they were sure of finding health workers. Most acknowledged that the situation was improving with establishment of specialised clinics especially at the national referral hospital, which run most days of the week.

*Previously I only saw a doctor once a month but now, I can see the doctor any day between Monday to Friday. *(1 Anthony_Male 1)

Some, however, were still hindered by lack of providers at health facilities.

* Doctors would come once in a month so I didn't have the vigor to go to hospital for care. *(16 Kabumba_female 1-*Mukono, Rural*)

Lack of personnel as a barrier to health seeking behaviour was mainly reported by patients from the rural areas. However, some also stressed that their district hospital operated a one-day clinic in a week for diabetes and hypertension which limited access to specialised health workers for hypertension. 

*Diagnostic Supplies*. Supply issues entailed the availability or lack of/or inadequacies in diagnostic equipment. It was common for patients in rural areas to report that they lacked a place where they could have their hypertension routinely monitored.

*… the clinics here in the village do not have the BP machines … all they do is to ask you; ‘what are you suffering from'? Then they base on what you have told them to give you a few tablets to relieve you from the pain. *(6 Bukamunye B_Female 2-*Buikwe, Urban*)

Another patient reported, “the biggest challenge we are facing as people with hypertension in this village is lack of somewhere we can go for emergency blood pressure monitoring” (41 Ssaza_female 3-* Mukono, Urban*).

*(2) High Burden of Acute Care at Health Facilities.* It was quite interesting to note that some patients did not seek care because they thought health workers would not attend to them since they [patients] did not have severe symptoms to warrant attention. This was especially in relation to those who desired regular BP monitoring. They said that hospitals were full of the severely ill meaning that a visit to health facilities for routine hypertension monitoring would be an additional burden to an already overwhelmed system. This subtheme was best illuminated by a patient (33 Malongwe Ajjija_male 2-*Buikwe, Urban*) who reported that it was difficult to just walk into a health facility and request a BP measurement. He said that such a daring would be considered luxurious because most of the facilities focus their efforts and resources on the very ill not the healthy looking individuals who walk in for BP measurements. The participant exemplified as follows:

*One challenge has been the monitoring of blood pressure … It's hard to just walk up to a health worker and tell them you would like to check your blood pressure … It's hard for the health worker to really give you attention … because you would have gone there for luxury and not as a patient. So when I would request for a blood pressure check-up; they would ask what the problem was. They cannot work on a person like me yet there are many patients who need help. *(33 Malongwe Ajjija_male 2-*Buikwe, Urban*)

*(3) Traditional Medicine*. With respect to the challenges surrounding access to modern health services, some patients resorted to using alternative medicines and seeking care from traditional practitioners. Most patients seeking complimentary medicines used herbs and some reported that they consulted traditional healers for their hypertension. Patients considered herbs easily accessible and to a certain extent comparably affordable. Planting them in the back yards enhanced access to herbs. In addition, most of the herbal remedies reported were readily available on the local market. However, it was also noted that some special herbs including those exported from abroad were sometimes difficult to access and expensive and that some traditional healers asked for certain items such as chicken and goats, which deterred patients from consulting them.

*At first I tried local medicine like mumbwa but when the traditional healers started asking goats from me I couldn't manage that because I didn't have the money to buy these goats so I stopped visiting traditional healers *(47 Vvuluga_female 1-*Buikwe, Urban*).* It was Chinese medicine; … my brother brought it for me. I drank it … in fact I improved slightly; the fast heart beats stopped and my head also stopped hurting; only that I failed to use it because of my finances. *(14 Jagary Cable_female 1-*Buikwe, Urban*)

It was also observed that traditional healers do not necessary stay in the same communities where they provide their services. In some cases, patients reported that they abandoned their local treatment remedies because their providers [traditional healers] were out of reach. This patient alluded as follows:

*From the time I would buy their medicine it would take like two to three months before they [traditional healers] returned to our village, this stopped me from using their medicine. *(16 Kabumba_female 1-*Buikwe, Urban*)

*(4) Perceived Provider Abilities and Behaviours and Overall Quality of Care*. This theme comprises the four subthemes: (1) patients' perceptions about their providers' abilities in terms of skills and knowledge competences; (2) perceived providers' attitudes towards work and the feeling of being treated with or without respect; (3) providers' advising role towards their patients' health seeking behaviours; and (4) perceived quality of care. 

*Patients' Perceptions about Their Providers' Abilities in Terms of Skills and Knowledge Competences.* Whereas most patients perceived their providers to have sufficient knowledge and the required skills to manage hypertension, some had reservations. Those who had reservations depicted their providers as not having sufficient competencies. They felt the providers focused on treating symptoms rather than conducting a proper disease diagnosis prior to treatment.

*… it is better for the health worker to know the disease they are treating by examining the patient, but for them [referring to health workers] they just dish [give] medicine to patients based on whatever disease the patients mentions … If you tell them [health workers]; ‘I have headache due to over sleeping or that I have headache because I have fever', that is it – then he/she [health worker] will give you tablets to treat that. *(6 Bukamunye B_Female 2*-Buikwe, Rural*)

Providers implicated with such incompetence were mainly those operating at the lower level health facilities especially drug outlets, pharmacies, and rural health facilities. On the other hand, most patients had confidence in medical doctors. They also felt that hospitals were better prepared in managing hypertension compared to private and lower health facilities. 

*Perceived Provider Attitudes towards Work and the Feeling of Being Treated with or without Respect.* Patients noted that providers exhibited different attitudes towards their work and their patients. They felt very comfortable seeking care from providers who were friendly. Some providers were implicated in having unwelcoming behaviours and negative attitudes towards patients. Examples of negative behaviours reported in this study included barking at patients, treating patients disrespectfully, and being unconcerned. Patients noted that it was common to find such behaviours in the public facilities. Most patients complemented private health facilities for their welcoming and caring attitudes. In fact, one patient stated that, in the private clinics, they are treated as customers which motivates them to seek care from the private clinics although he was also quick to speculate that the warm care was possibly because of the financial incentives [since patients pay for the service] and the strict leadership. Some patients also noted that they did not appreciate the decision making process that their providers followed while helping them to get appropriate remedy for their ailments. They felt that providers exhibited unnecessary delays, which resulted in negative health outcomes. One patient expressed dissatisfaction and wondered why the healthcare workers could not agree on the next course of action and refer her in a timely manner given that they were failing to diagnose and manage her condition.

*Nurse … ‘her case cannot be handled here' … ‘we have to refer her to Mulago hospital; she will die here.' She would continue to tell the others to allow me go to Mulago stressing that they cannot handle me … But there was a nurse who would prescribe medicine and tell me, ‘These will work for you' so that's how I was. I would always go back to them complaining of not sleeping well; it was like that every time; until I got the doctor from Mulago*. (37 Namengo Female 1-* Buikwe, Urban*)

*Providers' Advising Role towards Their Patients.* In spite of the reported challenges with some modern health care providers, patients were to a greatest extent likely to follow the advice they received from their providers. Certain patients said that they were using herbal remedies because they had been advised to do so by their healthcare providers. However, others said that they were not using herbal remedies because their healthcare providers advised them against arguing that concurrent use of herbal and modern medicine could result in contraindications and that the efficacy and effectiveness of such medicines were not established. On the other hand, some traditional practitioners were also discouraging their patients from using modern medicines arguing that they were not effective.

*I left Nagalaama [hospital] with some medicine and when I reached the herbalists' place they stopped me from taking modern medicine. *(17 Kabumba_female 2-*Mukono, Rural*)

Health care providers also advised patients on lifestyle modification. Some patients were advised on salt intake, dietary modification, physical activity, alcohol consumption, and smoking. But the patients were also keen on reporting that although they put effort in following their providers' advice, some of the advices were not contextually feasible.

*… he told me not to eat many things; and yet I cannot stop eating some of those that he mentioned; he told me not to eat ‘posho'; sweet potatoes … and told me not to eat one meal; which is difficult in a village. *(20 Kateete Female 1-*Mukono Rural*)

*Perceived Quality of Care.* This theme generated conflicting reports. Some respondents felt that government hospitals provided high quality services compared to private health facilities in spite of the high patient loads and the long waiting time. On the other hand, patients commended private health providers for being swift in service provision, although they had concerns with regard to provider competencies in private facilities. Meanwhile, the majority felt that modern medicine had an edge over traditional medicine, although some respondents noted that the quality of traditional medicine is improving especially in terms of practice, dosing, and packaging. 

*Patient Waiting Time at the Facility*. Patients noted that it was very stressful to spend the whole day at a health facility waiting to be treated. The long waiting time was attributed to (1) high patient volume vis-a-vis the limited number of health workers; (2) sluggishness and being unconcerned on the side of the healthcare workers, and (3) a lack of clear patient flow protocol that was characterised with favouritism and lack of respect.

*it is because by the time you go to the hospital very early in the morning, you were feeling unwell but in the process of queuing in the hospital, there can come someone who knows the doctor and the person overtakes you – one thing that is annoying because you come to the hospital after you have slept unwell and only to be disappointed – do you see such a thing? *(Njeru_Female 1* Buikwe, Rural*)

As a consequence, some patients stopped going to certain hospitals because of the long time they spend trying to get a service. Moreover, they reported that they had to cue at every service point (consultation room, laboratory room, and dispensing room). They felt very high workload and long waiting times were most prominent at public health facilities.

#### 3.2.2. Patient Related Factors

Another overarching theme influencing compliance and health seeking behaviour for hypertension was “patient related factors.” The subthemes building up this theme: (1) awareness about having the disease; (2) perceived severity or fear of the consequences of the disease; (3) beliefs about the effectiveness of the therapy; (4) concerns about adverse effects; and (5) the fear of dependency on long-term medication. 

*Patients' Awareness about Having the Disease.* Awareness “the state of being aware or having knowledge that one is hypertensive” played critical role in HSB. Some patients reported presenting with no symptoms at the time of diagnosis, whereas others reported mild or severe symptoms as depicted by the following patients.

*When I went to the first clinic, they checked me and they said that I was hypertensive – I couldn't believe it. I went to another clinic and they still measured my BP and they told me that I was hypertensive, I still didn't believe it. I went to the third clinic where they measured my BP again and they found me hypertensive and they even started me on the drugs to treat it. *(10 DFI_female 2-*Mukono, Urban*)

Another patient reported, “I did not have any symptom; I was like any other normal person. I did not have rapid heartbeats” (14 Jagary Cable Female 1), and another one also narrated, “I really think that I had been hypertensive for quite a long time though I was unaware” (41 Ssaza_female 3-*Mukono, Urban*).

Respondents felt that creating awareness was dependent on having the patients' blood pressure measurements taken at a health facility or during a community outreach. However, they noted that opportunities to have their blood pressure measurements at the facilities or outreach sites were scarce. Because of the limited opportunities, and the asymptomatic nature of hypertension, most patients got to learn about their hypertension after experiencing severe symptoms or presenting with signs of end organ damage such as stroke.

*I took long to know that I was hypertensive … One time in 2000, I was taking my agricultural produce to Kampala for sale, while travelling I got a ‘stroke' in the car. I was then taken to a KCC health facility in Kampala. They (health workers) checked and found that I had hypertension, so that's when it started. From that day I knew that I had hypertension. I went on receiving treatment up to this day. *(26 Kyamabale_male 1-*Buikwe, Rural*)

*Perceived Severity or Fear of the Consequences of the Disease.* Patients who had mild symptoms perceived themselves to be at low risk and were likely not to seek care for their hypertension.

*… here in the village as long as you are still able to walk, there is no need of you monitoring your BP because you have hope that it will subside and you will get back to normal *(6 Bukumunye B_female 2).* I had the disease, but because it was not very serious I kept postponing going to the health facility. (31 Lusozi_male 3-Buikwe, Urban)*

Because of the low risk perceptions, such patients mainly depended on self-medication to treat symptoms or embraced a do nothing scenario. On the other hand, patients who perceived their disease to be severe especially after experiencing life-threatening symptoms were more likely to seek care promptly from bigger hospitals and avoid self-medication

… after surviving death, I no longer self-treat or cover up an illness [delay diagnosis]. (41 Ssaza_female 3-Mukono, Urban)

*The Patient Beliefs about the Effectiveness of the Therapy or Recommended Advice.* Some patients' HSB and compliance were dependent on their perceptions about the effectiveness of the treatment or recommended provider advice. Effective remedies encouraged patients to return to their providers for refills, whereas patients who reported otherwise commonly reported trying out alternatives in search for better therapies.

*I have managed to treat this disease (Hypertension) for the many years I have lived with it. I have used various treatment methods. I first started with the modern method then later traditional medicine until now. However the traditional (medicine) has helped me more than the modern medicine. *(8 Bulamba_Bugungu_male 1-*Buikwe, Rural*)

Compliance based on perceived effectiveness of therapy applied both to modern as well as herbal medicine. Those who perceived herbal medicines to be effective in relieving their symptoms were likely to report usage of such remedies.

*I use the onions and the small egg plants – they help me a lot because whenever they bring them for me, I tend to feel better after using them *(34 Mayugwe Ssi_female 1-*Buikwe, Rural*).* It's very bitter and I know that hypertension can be controlled with bitter substances. When you take aloe vera, you feel well. *(6 Bulamba_Bugungu_ male 1-*Buikwe, Rural*)

Similarly, those who perceived modern medicines to be effective were likely to report modern medicine usage.

*Well I went to the hospital and they diagnosed me with hypertension and they started me on the drugs and later in the process of seeking care from the modern health facility, there was someone who advised me to also use the traditional herbal medicine which I used for a short time and later realized that it was useless. So I returned to the modern health facility and they gave me the drugs to take. *(P 16: Ngogwe_Kikoota_ female 1-*Buikwe, Rural*)

A third category reported using both. This category perceived that using both modern and herbal remedies had complimentary effectiveness and resulted in better health outcomes.

For the modern medicine, they can tell you to take one tablet a day (1 × 1) of which I have already taken it in the morning at around 8.00 AM. So by evening when I feel my BP raised due to the constant movements, that is when I take that Aloe Vera - it normalizes it and I finally sleep. (2 Bugga female 1-Buikwe, Rural)

*Patients' Concerns about Adverse Effects.* Patients reported that some therapies were not tolerable. They were associated with unpleasant outcomes. Some noted that adverse effects of some medicines were worse off than their hypertension. On the other hand, if the remedy was tolerable, usage was more likely. These concerns were reported for modern medicines as well as herbal medicines.

*In the beginning they introduced me to start using herbal medicine; but when I started using it I realized that it had bad side effects; my hands and legs were swelling. So I stopped and remained on the modern medicine (14 Jagary_cable_female 1-Buikwe, Urban). I do not like the [modern] medicine they provide; the medicine is bitter; others are tiny others are big. It makes ‘you' weak, and dizzy; even if ‘you' have a baby crying next to ‘you'; it's hard to carry the baby because ‘you' get weak. *(29 Lusozi_female 2-*Buikwe, Urban*)

*The Fear of Life Long Dependence on Medicine*. Lifelong dependence on antihypertensive was perceived as a burden and some patients could not believe that they were ready for such a course of action. As a result, some patients reported that they swallowed drugs occasionally to try and avoid over dependency on medicine in controlling their hypertension.

#### 3.2.3. The Socioeconomic and Structural and Cultural Environment

Besides, the healthcare and the patient related factors and several other issues were reported that influenced HSBs. We have summarized these factors under the socioeconomic and structural/cultural environment issues. More specifically, three topics were discussed: (1) education, information, and marketing of traditional medicine, (2) the patients' financial and social status, and (3) distance and transport.

*(1) Education, Information, and Marketing of Traditional Medicine.* Education, information, and marketing greatly influenced the use of alternative medicine for hypertension in this population. Patients reported that traditional practitioners, distributors, and proponents of herbal medicines embraced the power of sociomarketing, education, and active information sharing about their products. The most popular avenues of communication and promotion of herbal remedies included media houses such as radios and television, door-to-door information and drug distribution, and use of public address system in populated areas such as markets and assembly places.

*They give us the herbal medicine that is packed in the bottles and sold on the cars that pass by advertising the herbal medicine for the people to buy. They say that their medicines heal every sickness. So I buy those Rwenzori bottles with herbal medicine and take. *(23 Kikoota_female 1 –* Buikwe Rural*)

Another patient stated, “I would listen to him (traditional health practitioner) over the radio – that was before he went to the television; because up to date he is still on the radio and on the television. I listened to him and then I got his telephone contact and first I rang him and then I went there to see him” (11 DFI_male 1-*Mukono, Urban*).

On the other hand, no data was generated about efforts to use education, information, and marketing strategies to promote modern health care for hypertension. 

*(2) The Patient's Financial and Social Status.* Financial and socioeconomic status of the patients was critical in influencing compliance and HSBs of patients with hypertension. Specifically, financial and social aspects included the ability of the patients to meet their healthcare needs, family support, and advice from significant others. Most patients reported that hypertension was a lifetime disease that in the long run was too expensive. In fact some of them said they were already struggling with more pressing basic needs of life such as food and children school fees, which took precedence over their hypertension problems. For example, this patient narrated her story:

*… its money; because you may have the money and think you will go [to the hospital] at the end of the week and then there comes a person who needs the money more than you do, for example your child calls saying mammy send me money; there is this problem; so you decide to send the money to them; so you postpone and say I will go next week. So it gets to that; you later realize you spent two months without treatment. *(20 Kateete_female 1-*Mukono, Rural*)

On the other hand, those who had better financial standing were more likely to seek care for their hypertension.

*I don't have money problem to buy medicine. I have my work which earns me money that I use to buy medicine. *(1 Anthony male 1-*Mukono, Urban*)

In addition, patients with family members [children or other relatives] with better financial standing were reportedly supported to enhance their HSBs for hypertension.

*In fact my children and my brothers' money have been spent big time – most especially my son's money has been spent so much because he wishes me to get better but all in vain. *(Mayugwe_Female 2-*Buikwe, Rural*)

Family members also provided patients with psychosocial and moral support as well as reminding them about their scheduled appointments, drug refills, and taking the medicines. Moreover, acquaintances such as friends, community members with similar challenges, or community resource persons such as religious leaders were also reported. These particularly played a key role in providing guidance to patient on whether to use modern or traditional medicine. 

*(3) Distance and Transport.* The study shows that transport, distance, and related costs influenced health seeking behaviour of patients for hypertension. Patients from far-off areas (mainly rural) incurred higher costs of transport compared to their counterparts from urban neighbourhoods and those near the health facilities. Moreover, means of transport were not always reliably available in some settings. Consequently, rural residents commonly reported transport as a key barrier to seeking care, including adhering to scheduled hospital appointments.

*The only challenge I faced was lack of transport; the place was really far so it was hard to go there on foot. *(P 8: 16 Kabumba_female 1-*Mukono Rural*)

On the other hand, urban resident either did not mention transport as a barrier or they reported ease of access to health facilities as a motivator to seek care for their hypertension.

*It's because I am near the hospital so I don't spend on transport to go to Kawolo hospital. I just walk to the hospital. *(P 6: 14 Jagary_cable_ female 1-*Mukono, Rural*)

## 4. Discussion

The study shows that most patients in this setting utilise varied channels of care for their hypertension. In spite of the varied health seeking behaviours (HSBs), 8 in 10 patients interviewed in this qualitative study had uncontrolled hypertension. Insufficient hypertension control is prevalent in most of Sub-Saharan Africa [13] and data suggest that these high levels of uncontrolled hypertension are attributed to noncompliance, difficulty in obtaining medication, poverty, and poor access to health care [14–16]. In addition, health system deficiencies such as lack of antihypertensive medication and long distance to facilities have been implicated in affecting HSBs and control of hypertension [13, 17, 18]. Similarly, our study found that a range of health system challenges such as inadequate services, high cost of services and medicines, frequent drug stock outs, and quality concerns were strongly attributed to noncompliance and poor HSB patterns for hypertension.

In addition, HSBs were influenced by awareness, perceived severity, perceived effectiveness of recommended intervention/therapy, concerns about adverse effects, and fear of long-term dependence on medication. The influence of awareness on HSBs for hypertension is well established [11, 32]. Being aware of one's hypertension status is critical in initiation of care and impacts on self-care management practices [32] and is associated with reduced hypertension related complications and mortalities. However, availability and access to BP monitors were reported as a challenge in this study. However, perceived disease severity was critical in determining treatment compliance and HSB. In fact some of the patients reported that it was not necessary for them to continue with treatment or go to hospital if they felt well. On the other hand, manifestation of symptoms enhanced their compliance and HSBs. With mild symptoms, they mostly self-treated with herbal or modern medicines or consulted with providers at lower level health facilities including drug shops and pharmacies, whereas patients with severe symptoms reportedly sought care from hospitals or higher level health centers. The behaviours of these patients are consistent with those of the Koreans in the stroke study whose choice of care depended on their perceived severity of symptoms. For those who perceived themselves to have mild symptoms, they used over-the-counter drugs and folk remedies or visited health care facilities such as drugstores, public health agency, herbal clinic, or hospital [33]. These findings confirm the assertion that signs and symptoms influence people to seek diagnosis and treatment. The patients contemplate about the symptoms and then decide to take appropriate actions according to their perceived degree of disease severity [33]. When they perceive that the disease is very severe, they seek more specialised care but if they feel that the disease is less severe, they embark on self-care to try and resolve the discomfort.

HSBs were also influenced by the perceived effectiveness of the recommended treatment advice and this factor applied to modern as well as herbal alternative usage. Similar findings have been reported before [34–36]. In our previous study, use of traditional medicine was associated with the perception that herbal medicines were effective [34] and in Nigeria, patients strongly considered herbal medicines as viable alternative for a cure for their hypertension [37].

Another key factor was experiencing adverse reactions. Some patients reported that they experienced terrible side effects which were regarded worse off than their hypertension. Likewise, patients in Nigeria stopped medication as a result of experienced side effects [36]. Additional studies in Nigeria [11, 36], Democratic Republic of Congo [14], and Vietnam [3] have reported similar findings.

Health seeking behaviour was to a greater extent also influenced by the socioeconomic status of patients and structural and cultural factors. Patients with better socioeconomic standing had better compliance and better health seeking behaviour. The former were more likely to afford both the direct and indirect costs of health care, whereas the later, even basic amenities of life such as food, was sometimes a problem. Osamor argued that when people are hungry, nothing else matters to them except food [36]. Similar findings related to socioeconomic status and its influence on compliance and health seeking behaviour have been reported in other settings [38, 39]. In South Africa, authors reported that patients with greater economic resources were more likely to seek treatment from private doctors and spend considerably more for all types of health services compared to their counterparts in the low socioeconomic index [39]. Likewise in Nigeria, poor socioeconomic status, low level of education, unemployment, lack of effective social support networks, unstable living conditions, long distance from treatment center, and high cost of transport negatively influenced treatment compliance and health seeking behaviours for hypertension [38].

## 5. Implications for Policy and Practice

These findings have significant policy and practical implications on the delivery of services for hypertension. These revolve around the need for educating both the patients and the general population on hypertension, conducting routine screening, ensuring continuity of care, adherence to treatment, patient provider relations, organisation of services to minimize multiple movements across service points, availing services, and enhancing competences of staff.

Although the multiple channels of care for hypertension increase the reach of the services, the channels make it difficult for providers to monitor treatment success rates. Nonetheless, it is unlikely that the patients' behaviour of seeking care from various channels will stop in the near future as long as the comparative advantages of the different care pathways remain. For example, self-medication is comparatively cheaper and saves a lot of patients' time. Yet, it denies the patient the opportunity to receive professional management of the disease which may result in disease complication due to poor management. Therefore, empowering lower level facilities including private drug outlets through training, support supervision, and regulations may be harnessed to reduce risks of self-medication and enhance screening and monitoring, drug refills, and referral compliance. This empowerment would also reduce the challenges related to distance and access especially among the rural folks. On the other hand, higher level facilities should progressively be exempted from routine screening and monitoring which contributes to high patient volumes but instead be equipped and motivated to deal with referral cases in a timely manner. Meanwhile, it is also desirable to enhance the integration of traditional health practice with modern practice to bridge the gaps. Such integration would reduce bickering and situations where traditional health practitioners advice patients not to use prescribed modern medicines. Moreover, increased integration would increase interest in understanding traditional medicine and exploring its efficacy and effectiveness. With the increasing burden of chronic disease, a more sustainable financing mechanism is also warranted. A life time disease like hypertension in the long run becomes unaffordable even to the middle income category in this population. Hence, noncompliance and poor seeking behaviour and related negative health outcomes are inevitable. Exploring feasibility of instituting insurance schemes for chronic disease is recommended.

The patient related barriers have also significant implications. Jin and colleagues recommend that therapy related concerns should be addressed to contribute positively in improving patient's compliance. Prescribing medication with noninvasive route of administration and simple dosing regimens might motivate patients to be compliant [40]. Long duration of treatment period and medication side effects might compromise patient's beliefs about medication effectiveness. Therefore, healthcare providers should consider therapy related problems when designing the therapy plan and involve the patients in the process to minimize the possible therapeutic barriers [40].

## 6. Strengths and Limitations

This qualitative study highlights the factors influencing compliance and health seeking behaviour for hypertension among aware hypertensive patients in Mukono and Buikwe Districts in Uganda. The strength of the study lies in accruing the patients in their natural environment and learning from their experiences by using interviews in the community at the patients' homes. The patients were purposively selected to obtain a varied sample comprising of different gender, residential status, and district of residence. Limited transferability can be claimed given the small scale sized nature of the study which was conducted in only two districts in Uganda. Despite these limitations, the findings are insightful and of practical importance for programs and intervention trials aimed at feasible strategies to lower hypertension in this population.

## 7. Conclusions

Hypertension control among aware hypertensive patients in this setting is very low with eight in ten of patients aware of their hypertension not controlled. These control levels are attributable to poor compliance and health seeking behaviours which in turn are influenced by a number of factors which are closely related to the health system, the patient, and the socioeconomic circumstances and the culture of using traditional medicines. Strategies are needed to improve hypertension control in this population. Such strategies should leverage the opportunities at the various levels and traditions of care and address the barriers to health seeking behaviours.

## Figures and Tables

**Table 1:** 

Characteristics	Number
Sex	
Male	17
Female	31
Residential status	
Rural	24
Urban	24
Education status	
Primary or less	25
Post primary	23
Marital status	
Single	13
Married	35
Age	
Overall median age	54
Median age (female)	51.5
Median age (male)	54
Measured BP on the interview date	
(SBP < 140 mmHg and DBP < 90 mmHg)	8
Stage 1 (SBP = 140–159/ DBP = 90–99)	20
Stage 2 (SBP = 160–179/ DBP = 100–109)	11
Stage 3 (SBP ≥ 180/DBP ≥ 110)	9

## Data Availability

All data relevant for this data has been included in this manuscript.

## Appendix

### Factors Influencing Health Seeking Behaviours of Hypertensive Patients in Mukono and Buikwe in Uganda

*Qualitative Interview Guide*

1. For how long have you had (known that you have) hypertension?
2. What are you're own thoughts about the cause of your hypertension?
3. How did you get to know that you had hypertension?
  - Probe for symptoms and diagnosis.
  - How did you deal with those symptoms before you got diagnosed?
    - Probe for things the patient did before he/she got actually diagnosed with hypertension (selftreatment, traditional healers,…)
  - How and when did you decide to seek treatment, what changed?
    - Probe for change in symptoms, or things that might have encouraged them to actually go and seek help
    - How did they actually seek help, who helped them in making that decision where to go
    - how was the process of seeking help (meaning did you see many different people before actually seeing a doctor, how did you become diagnosed with hypertension)
4. Can you explain the care and treatment process from the time you discovered that you are hypertensive?
  - Probe for access to treatment and care (traditional healers, drug shops, self medication with herbs or modern medicine, health care facilities)
    - Probe for barriers and facilitators
  - frequency of blood pressure monitoring and who monitors this?
  - Who helps you deal with your hypertension (family, neighbours, Village health team member (VHT))
  - What do they help you with?
  - Adherance to treatment and defaults.
5. Since you became hypertensive, have you used any other methods to treat your hypertension?
  - Probe for herbs or other things that they might have tried
    - If yes, where do you get them from?
    - Does that interfere with your other medication (if used)
    - Why do you use herbs for your hypertension?
    - Can you explain how you came to use these products (who introduced you to these methods)
6. Since you became hypertensive, have you visited or consulted a traditional healer for your hypertension?
  - Probe for advice and treatment received, experience with the treatment or advice
  - What influenced your decision to go to the traditional healer for your hypertension?
  - How were you treated?
7. Since you became hypertensive, have you been to a modern healthcare facility (health center, hospital or private clinic) for your hypertension? If yes:
  - Probe for treatment and advice recieved, level of satisfaction
  - What influcend your decision to go to a modern healthcare facility for your hypertension?
  - How where you treated?
8. What is your preferred healthcare service provider (modern or traditional)? Explain your preference, why do you choose one over the other or both and why?
9. What recommendations would you make that can help hypetensive patients to seek care from modern healthcarefacility?

We are now at the end of the interview and the questions I wanted to ask you. Are there any other issues you find important regarding this topic that we did not talk about? What?

Thank you for your valuable time and accepting to participate in this study.
